# The Effect of Laboratory Parameters on the Hospital Stay and Mortality in Pediatric Patients Who Underwent Lobectomy and Pneumonectomy

**DOI:** 10.3390/medicina61010026

**Published:** 2024-12-28

**Authors:** Ahmet Ulusan, Hilal Zehra Kumbasar Danaci, Bekir Elma, Maruf Sanli, Ahmet Feridun Isik

**Affiliations:** 1 Department of Thoracic Surgery, Gaziantep University, 27310 Gaziantep, Turkey; drbekirelma@gmail.com (B.E.); sanlimaruf@yahoo.com (M.S.); abayboraa@msn.com (A.F.I.); 2 Department of Thoracic Surgery, Alanya Alaaddin Keykubat University Training and Research Hospital, 07400 Alanya, Turkey; zehra_kumbasar_53@hotmail.com

**Keywords:** thoracic surgery, lobectomy, pneumonectomy, pan-immune-inflammation value, neutrophil-to-lymphocyte ratio, platelet-to-lymphocyte ratio

## Abstract

*Background and Objectives:* This study aims to evaluate the prognostic significance of various laboratory parameters in predicting the length of hospital stay and mortality among pediatric patients undergoing lobectomy and pneumonectomy for infectious or noninfectious diseases. *Materials and Methods:* This study was conducted by retrospective data analysis of 59 pediatric patients who underwent lobectomy and pneumonectomy due to variable diseases at the department of chest surgery. Pediatric patients diagnosed with variable diseases and who underwent lobectomy or pneumonectomy, patients who were hospitalized during the study period and underwent surgical intervention, and patients who had at least one laboratory parameter recorded before surgery were included in the study. Laboratory parameters included white blood cell (WBC) count, red cell distribution width (RDW), platelet (PLT) count, neutrophil count, mean platelet volume (MPV), monocyte count and lymphocyte count. Laboratory results were obtained from the patients’ most recent blood samples before surgery. In addition, derived ratios such as platelet-to-lymphocyte ratio (PLR), MPV-to-PLT ratio (MPR), MPV-to-monocyte ratio (MMR), MPV-to-lymphocyte ratio (MPVLR), monocyte-to-lymphocyte ratio (MLR) and neutrophil-to-lymphocyte ratio (NLR), and Pan-Immune-Inflammation Value (PIV) were calculated and their potential prognostic values were evaluated. *Results:* The mean age of the patients was 6.3 years (0–15 years). The gender distribution of the patients in the study group was determined as 36 male patients (61%) and 23 female patients (39%). Lobectomy was the most commonly performed surgical procedure, accounting for 83% of cases, while pneumonectomy was applied in 17% of cases. The postoperative complication rate was observed as 34%. The mean hospital stay of the patients was calculated as 12.61 days (2–84 days), while the mortality rate was recorded as 8.5%. The relationship between the neutrophil count and the length of hospital stay is quite strong, and the r value was calculated as 0.550 (*p* < 0.01). PIV showed a strong correlation with length of hospital stay, with an r value of 0.650 (*p* < 0.01). A significant relationship was also found between PLR and length of hospital stay (r = 0.500, *p* < 0.01). The parameters with the highest positive effect on the hospital stay are neutrophil count (B = 0.400, *p* < 0.01) and PIV (B = 0.500, *p* < 0.01). The mean hospital stay was 10.00 days (2–30) in patients without complications and 15.00 days (5–40) in patients with complications, and this difference was also significant (*p* < 0.01). Finally, the mortality rate was 2.5% in patients without complications, while it was determined as 20% in those with complications (*p* < 0.01). *Conclusions:* This study determines the effects of laboratory parameters (particularly PIV, neutrophil, PLR, and NLR) on the duration of hospital stay, development of complications, and mortality and emphasizes the importance of monitoring these parameters in the treatment of infectious or noninfectious diseases.

## 1. Introduction

Thoracic surgeries, including lobectomy and pneumonectomy, are critical interventions used to treat both infectious and noninfectious diseases of the lungs. In pediatric populations, these procedures are often performed for conditions such as chronic infections, congenital malformations, and lung tumors. Infectious diseases, such as empyema and severe pneumonia, may necessitate the removal of affected lung tissue when conservative treatments fail to resolve the infection or prevent complications like abscess formation. On the other hand, noninfectious diseases, including congenital cystic adenomatoid malformations (CCAM) or pulmonary sequestrations, often require surgical intervention to prevent future complications, such as recurrent infections or respiratory failure. Despite their potential to improve patient outcomes, thoracic surgeries carry significant risks, particularly in children, due to the potential for postoperative complications and prolonged recovery times. As such, careful preoperative evaluation and postoperative monitoring are essential to minimize the risks and ensure better outcomes [1,2,3,4].

Despite advancements in surgical techniques and perioperative care, managing patients with infectious or noninfectious diseases remains challenging due to complications such as prolonged hospital stays and increased mortality. Surgical treatment, including procedures like lobectomy and pneumonectomy, is the primary approach for managing infectious diseases by removing infected tissue and controlling the spread of the infection [5,6]. However, outcomes such as hospital stay duration and mortality rates vary based on multiple factors.

Postoperative monitoring of patients, particularly through the evaluation of laboratory parameters, plays a critical role in determining prognosis and treatment success, helping to guide clinical decisions and improve patient management. Studies have highlighted the significance of inflammatory markers such as white blood cell (WBC) count, platelet (PLT) count, and various hematological ratios in predicting clinical outcomes in different surgical and non-surgical contexts [7]. Parameters such as the neutrophil-to-lymphocyte ratio (NLR), platelet-to-lymphocyte ratio (PLR), and mean platelet volume (MPV) have shown promise as prognostic markers in various diseases [7].

In recent years, the role of laboratory parameters in predicting hospital stay duration and mortality rate outcomes has gained attention, yet studies specifically focusing on the correlations between these parameters and outcomes in the context of lobectomy and pneumonectomy for infectious or noninfectious diseases are limited. This study aims to evaluate the prognostic significance of various laboratory parameters in predicting the length of hospital stay and mortality among pediatric patients undergoing lobectomy and pneumonectomy for infectious or noninfectious diseases.

## 2. Materials and Methods

### 2.1. Study Design and Study Population

This study utilized a retrospective design, analyzing data from 59 pediatric patients who underwent lobectomy or pneumonectomy at the Department of Chest Surgery, Faculty of Medicine, Gaziantep University, between 2015 and 2024. This design aligns with the research objective of evaluating relationships between laboratory parameters and clinical outcomes in pediatric thoracic surgeries.

Inclusion Criteria:

Pediatric patients were included if they:

1. Were diagnosed with infectious or noninfectious diseases requiring lobectomy or pneumonectomy.
2. Were hospitalized during the study period and underwent the surgical intervention.
3. Had at least one recorded laboratory parameter prior to surgery.

Exclusion Criteria:

Patients were excluded if they:

1. Had other malignancies or serious systemic diseases unrelated to the study objectives.
2. Developed significant complications during surgery that prevented further monitoring.
3. Were hospitalized for surgical procedures other than lobectomy or pneumonectomy.
4. Had incomplete or missing preoperative laboratory data. No data imputation was performed to maintain dataset integrity and reliability.

The sample size of 59 patients represents the total eligible cases during the study period and was constrained by the rarity of pediatric lobectomy and pneumonectomy procedures. A post hoc power calculation was conducted to evaluate the statistical power of the analyses. While the sample size limits generalizability, it provides valuable preliminary insights into this specific population. Future multicenter studies with larger cohorts are needed to confirm these findings and enhance external validity.

Demographic data, pre-operative and post-operative laboratory findings, and surgical outcomes of the patients included in the study were reviewed. Laboratory data were collected retrospectively from the hospital’s electronic medical record system. All blood samples were analyzed in the same laboratory at the Department of Chest Surgery, Gaziantep University, using standardized automated equipment to ensure consistency across measurements. The laboratory followed strict internal quality control protocols to maintain reliability. Blood samples were collected within 24 h before the surgical procedure to minimize variability in preoperative inflammatory and hematological parameters. To ensure reproducibility, the data collection process was supervised by experienced personnel, and any discrepancies in the recorded values were cross-verified with the original patient files. Laboratory parameters included white blood cell (WBC) count, red cell distribution width (RDW), platelet (PLT) count, neutrophil count, mean platelet volume (MPV), monocyte count, and lymphocyte count. Laboratory results were obtained from the patients’ most recent blood samples before surgery. In addition, derived ratios such as platelet-to-lymphocyte ratio (PLR), MPV-to-PLT ratio (MPR), MPV-to-monocyte ratio (MMR), MPV-to-lymphocyte ratio (MPVLR), monocyte-to-lymphocyte ratio (MLR), and neutrophil-to-lymphocyte ratio (NLR) were calculated, and their potential prognostic values were evaluated. The Systemic Inflammatory Index (SII) was calculated by total number of neutrophils × total number of platelets/total number of lymphocytes. The Pan-Immune-Inflammation Value (PIV) was computed by multiplying the SII value and monocyte count [8].

The duration of hospital stay of the patients was monitored throughout the period after surgery, mortality rates were recorded during the hospital stay of the patients, and the relationship between these data and laboratory parameters was analyzed.

### 2.2. Statistical Analysis

Statistical analysis was performed using SPSS software, version 27.0 (SPSS Inc., Chicago, IL, USA). Quantitative data were described as medians (Min–Max), and categorical data were presented as frequencies and percentages. Pearson correlation analysis was conducted to assess the strength and direction of the relationships between laboratory parameters and key outcomes, such as the length of hospital stay and mortality. The statistical significance of the correlations was determined at *p* < 0.05. To address potential multicollinearity among predictors, we conducted Variance Inflation Factor (VIF) analysis prior to performing regression analyses. Variables with a VIF value >10 were considered to exhibit significant multicollinearity and were either removed or combined with related predictors. This ensured the reliability of the regression models and avoided overestimation of predictor effects. Linear regression analysis was used to examine the relationship between preoperative laboratory parameters and the length of hospital stay. The dependent variable was the length of hospital stay, and independent variables included hematological parameters and derived ratios (e.g., NLR, PLR, and PIV). For mortality analysis, logistic regression models were utilized. Regression results were presented with B (Unstandardized), Beta (Standardized), t values, and *p* values. Statistical significance was set at *p* < 0.05. Derived ratios were calculated using validated laboratory results. To minimize computational errors, each calculation was verified by two independent researchers. Ratios such as PIV were computed using established formulas, including neutrophil × platelet/lymphocyte for SII and multiplying SII by monocyte count for PIV.

## 3. Results

Sociodemographic, laboratory, hospital stay, and mortality findings of the patients in the study are shown in Table 1. The mean age of the patients was 6.3 years (0–15 years). The gender distribution of the patients in the study group was determined as 36 male patients (61%) and 23 female patients (39%). Lobectomy was the most commonly performed surgical procedure, accounting for 83% of cases, while pneumonectomy was applied in 17% of cases. The postoperative complication rate was observed as 34%, and the most common of these complications were postoperative infections (10.2%). In addition, bleeding (5.1%), pulmonary complications (8.5%), reactional pneumonia (3.4%), and suture site complications (6.8%) were reported. Among the laboratory parameters, the mean WBC count was 11.46 (5.25–40.72), RDW was 15.35% (11.9–20.3), PLT count was 385.04 (77–704), neutrophil count was 6.38 (1.4–22.15), MPV was 9.76 (7.5–11.1), monocyte count was 0.90 (0.41–2.94), and lymphocyte count was 3.99 (0.82–14.4). In addition, PIV was determined as 50 (20–100), (PLR) as 130 (90–170), MPVPR as 0.11 (0.08–0.15), MPVMR as 10 (5–15), MPVLR as 2.5 (1.5–3.5), MLR as 0.27 (0.15–0.40) and NLR as 2.5 (1.5–3.5). The mean hospital stay of the patients was calculated as 12.61 days (2–84 days), while the mortality rate was recorded as 8.5% (Table 1).

The correlation between the length of hospital stay and mortality findings of the patients and laboratory findings is shown in Table 2. A positive correlation was found between WBC and the length of hospital stay, and the r value was determined as 0.450 (*p* < 0.01). The RDW also had a significant correlation with the length of hospital stay, and the r value was determined as 0.350 (*p* < 0.05). The PLT count showed a negative correlation of −0.400 (*p* < 0.01). The relationship between the neutrophil count and the length of hospital stay is quite strong, and the r value was calculated as 0.550 (*p* < 0.01). The relationship between the MPV and the length of hospital stay is weak, and the r value was determined as 0.200 (*p* > 0.05). A significant relationship was also found between monocyte count and length of hospital stay, with an r value of 0.250 (*p* < 0.05). Lymphocyte count was not found to have a significant relationship with length of hospital stay (r = −0.100, *p* > 0.05). PIV showed a strong correlation with length of hospital stay, with an r value of 0.650 (*p* < 0.01). A significant relationship was also found between PLR and length of hospital stay (r = 0.500, *p* < 0.01). No significance was shown between MPVPR, MPVMR, and MPVLR and length of hospital stay. Finally, a significant relationship was found between MLR and length of hospital stay, with an r value of 0.250 (*p* < 0.05). NLR had a positive relationship with length of hospital stay, with an r value of 0.550 (*p* < 0.01) (Table 2).

**Figure 1:**
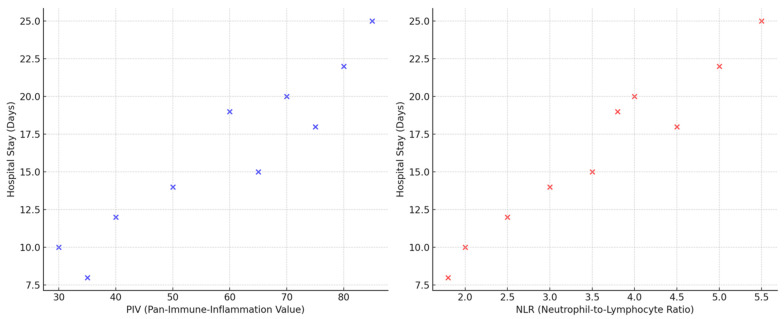
Correlations of PIV and NLR with hospital stay.

The linear regression analysis of the patients’ hospital stay and laboratory findings is shown in Table 3. The parameters with the highest positive effect on the hospital stay are neutrophil count (B = 0.400, *p* < 0.01) and PIV (B = 0.500, *p* < 0.01). While the increase in the neutrophil count contributes to the prolongation of the hospital stay, a high PIV indicates an increase in systemic inflammation and therefore a prolongation of the hospital stay. A positive relationship was found between the WBC count and the hospital stay (B = 0.300, *p* < 0.01), and the red cell distribution width (RDW) (B = 0.250, *p* < 0.05) also showed a similarly significant relationship. A negative correlation was found with the PLT count (B = −0.250, *p* < 0.01), indicating that high PLT levels have a prolonging effect on the duration of hospitalization. There was no significant correlation between MPV and duration of hospitalization (B = 0.150, *p* > 0.05), but a significant correlation was found with monocyte count (B = 0.200, *p* < 0.05). Lymphocyte count showed a negative correlation (B = −0.100, *p* > 0.05), which was not statistically significant. On the other hand, parameters such as PLR (B = 0.300, *p* < 0.01) and MPVMR (B = 0.250, *p* < 0.01) have prolonging effects on the duration of hospitalization (Table 3 and Figure 1).

The comparison of the findings with the development of complications in the patients is shown in Table 4. The mean WBC was found to be 10.50 (5.25–15.00) in patients without complications and 13.20 (8.00–40.72) in patients with complications and this change is statistically significant (*p* < 0.01). While RDW was 14.50% (11.0–18.0), it was found to be 16.80% (12.0–20.0) in those with complications and this is also significant (*p* < 0.05). PLT was determined to be 380.00 (150–600) in those without complications and 310.00 (200–500) in those with complications, and a significant difference was observed (*p* < 0.05). While the neutrophil count was 5.20 (1.4–10.0) in patients without complications, it reached 8.80 (3.0–22.15) in patients with complications, and this change was statistically significant (*p* < 0.01). Similar significant differences were observed in other laboratory parameters such as monocyte count and PIV. The mean hospital stay was 10.00 days (2–30) in patients without complications and 15.00 days (5–40) in patients with complications, and this difference was also significant (*p* < 0.01). Finally, the mortality rate was 2.5% in patients without complications, while it was determined as 20% in those with complications (*p* < 0.01). These findings emphasize the effects of laboratory parameters on the development of complications (Table 4).

## 4. Discussion

This study aimed to evaluate the effects of laboratory parameters on the length of hospital stay and mortality in pediatric patients who underwent lobectomy and pneumonectomy due to infectious or noninfectious diseases. The findings obtained in our study, when compared with similar studies in the literature, indicate that laboratory parameters have an important prognostic value on clinical outcomes. In particular, high values of parameters such as WBC, PLT, neutrophil, MPV and PIV, PLR, and NLR were associated with prolonged hospital stay and an increased risk of mortality.

In our study, the finding of a significant relationship between the length of hospital stay and laboratory parameters emphasizes the role of parameters such as WBC, PLT, neutrophils, PIV, MPV, and RDW in the postoperative period. In particular, an increase in the PIV and neutrophils count was associated with a longer hospital stay. This finding indicates an increase in inflammation and the risk of infection, which causes patients to stay in the hospital for a longer period. Similarly, the study by Gulmez et al. indicated that a high neutrophil count was associated with postoperative complications [9]. In addition, they found that high WBC and PLT levels were also associated with postoperative complications. High WBC values indicate an increased risk of systemic inflammation and infection, while increased PLT indicates an increased risk of thrombosis, which may prolong the recovery time of patients. Additionally, studies by Souza et al. and Zhang et al. have shown that high MPV levels are associated with prolonged hospital stays in surgical patients. The authors noted that MPV is a marker of inflammation and therefore high MPV values may be associated with longer hospital stays [10,11]. Kumar et al.’s study examined the effect of high levels of RDW on complications and length of hospital stay after pediatric cardiac surgery. They stated that high RDW values were associated with more complications in the postoperative period and prolonged hospital stay [12]. A limited number of studies emphasize that the PIV value is highly correlated with the duration of hospital stay and severity of disease in patients receiving post-surgical treatment [8,13,14,15,16,17]. This suggests that monitoring laboratory findings after surgery may help improve the treatment process and make more effective decisions in the clinical management of patients.

The relationships between mortality rates and laboratory parameters are also noteworthy. In our study, it was observed that inflammatory markers such as Neutrophil, PIV, PLR, and NLR showed a significant correlation with mortality. High PIV indicates increased systemic inflammation and therefore increased mortality risk. Bilgin et al. reported that high PIV values were found to be associated with poor prognosis [14]. In a study involving laparoscopic colectomy patients, Song et al. found that an elevated PLR on postoperative day 3 was associated with failure of enhanced recovery after surgery (ERAS) [18]. Feng et al. reported that higher PLR values correlated with active ulcerative colitis, suggesting its potential as a marker for disease activity [19]. Shi et al. reported that high PIV, NLR, MLR, and PLR values are associated with poor prognosis in patients who have undergone laryngeal and pharyngeal tumor surgery [20]. Similarly, Shen et al. showed that high PLR values increased the risk of mortality in patients with acute respiratory infection [21]. In addition, studies by Xie et al. and Zhai et al. reported that high PLR values had negative effects on overall mortality and this was observed in various disease groups [22,23]. In a limited number of studies in which lobectomy and pneumonectomy surgery was performed, it was reported that NLR, MLR, and PLR values had negative effects on the length of hospital stay and overall mortality, and this situation was observed in various disease groups [24,25,26,27,28].

Postoperative complications prolong the hospital stay and increase mortality rates. Bagheri et al. report 10 years of surgical experience on 78 patients with pulmonary infectious diseases [29]. Postoperative complications occurred in 23% of their patients. The most common complication was air leakage. In addition, complications such as fistula, empyema, seroma, and wound infection were also reported. They compared the surgical outcomes and postoperative complications between 3 different surgical methods: the Ugon method, cystectomy, and lobectomy. It was stated that cystectomy and lobectomy had lower complication rates than the Ugon method [29]. Behnia et al. studied 32 hydatid cyst patients and compared cystectomy and lobectomy surgery. They stated that cystectomy surgery was more preferable than cyst surgery in terms of length of surgery, length of hospitalization, and postoperative complications, although not statistically [30]. Yekeler et al. examined the cases in which lobectomy was performed for the treatment of pulmonary hydatid cysts in a study covering 36 patients. Complications (Parenchymal destruction, hemoptysis, and parenchymal loss) were observed at a rate of 25%. No deaths were reported [7]. In our study, lobectomy (83%) and pneumonectomy (17%) were successfully performed as surgical methods. Postoperative complications were observed at a rate of 34%. The most common of these complications were postoperative infections (10.2%), followed by bleeding (5.1%), lung complications (8.5%), reactional ventilation (3.4%) and suture site complications (6.8%). The mortality rate in patients with complications was 20%.

It was stated in the literature that postoperative complications are a common complication after surgical interventions, especially in pediatric patients, and their management is difficult [31,32]. In the study by Gulmez et al., preoperative and postoperative blood values of 37 pediatric pulmonary hydatid cyst patients were analyzed. They reported that WBC, eosinophil, neutrophil, eosinophil-to-lymphocyte ratio (ELR), and systemic immune inflammatory index (SII) values increased statistically significantly in patients with complications [9]. In our study, WBC, RDW, PLT, neutrophil, monocyte, and PLT levels showed a significant increase in patients who developed complications. There was a significant increase in other laboratory parameters such as PIV, PLR, MLR, and NLR levels in patients who developed complications. While the hospital stay was shorter in patients without complications, it was prolonged in those with complications. Finally, the mortality rate showed a significant increase in patients who developed complications. This situation once again emphasizes the importance of postoperative monitoring and infection control in surgical interventions due to infectious diseases such as tuberculosis, bronchiectasis, and hydatid cyst.

### Limitations of the Study

This study has several limitations that should be considered. First, it employed a retrospective design, which may introduce biases related to data collection and interpretation, limiting the ability to control for confounding variables. The sample size of 59 patients, while providing valuable preliminary insights, reflects the rarity of pediatric lobectomy and pneumonectomy procedures, which are inherently specialized surgeries with limited eligible patient populations. This constraint is common in studies focusing on rare and complex surgical interventions. Additionally, being a single-center study restricts the applicability of findings to other settings with different patient populations or treatment protocols. The variability in surgical techniques between lobectomy and pneumonectomy may also influence outcomes, complicating direct comparisons. Furthermore, the impact of specific complication types (e.g., infections vs. bleeding) on laboratory parameters was not fully explored, which may limit the interpretation of their predictive value. Despite these limitations, the study contributes valuable insights into the role of laboratory parameters in predicting outcomes in pediatric patients with infectious or noninfectious diseases, highlighting the need for further research to confirm these findings.

## 5. Conclusions

In conclusion, this study demonstrates the effects of laboratory parameters (particularly PIV, neutrophil, PLR, and NLR) on the duration of hospital stay, development of complications, and mortality, and emphasizes the importance of monitoring these parameters in the treatment of infectious or noninfectious diseases in pediatric surgery. These parameters may serve as useful tools for identifying patients at higher risk of prolonged hospitalization or adverse outcomes. Future studies should support these findings and shape treatment approaches by providing similar analyses in larger patient groups. Future research with larger, multicenter cohorts and prospective study designs is needed to validate these results and clarify the role of laboratory parameters in clinical decision-making. By addressing these limitations, subsequent studies can better determine how laboratory markers contribute to risk stratification and guide targeted management strategies in pediatric thoracic surgery.

## Figures and Tables

**Table 1:** Sociodemographic, laboratory, hospital stay, and mortality findings of the patients in the study.

Parameters	Patients (N = 59) N (%) Median (Min–Max)
Gender	
Male	36 (61%)
Female	23 (39%)
Age (years)	6.3 (0–15)
Diseases	
Hydatid cysts	11 (19%)
Congenital (CCAM)	15 (25%)
Malignancy	5 (8%)
Suppurative infections (Most of Tuberculosis and Bronchiectasis)	27 (46%)
Trauma	1 (2%)
Surgery	
Lobectomy	49 (83%)
Pneumonectomy	10 (17%)
Complications	20 (34%)
Postoperative infection	6 (10.2%)
Bleeding	3 (5.1%)
Pulmonary complications	5 (8.5%)
Postoperative respiratory distress	2 (3.4%)
Suture site complications	4 (6.8%)
WBC (×10^3^/µL)	11.46 (5.25–40.72)
RDW (%)	15.35 (11.9–20.3)
Platelet (PLT) (×10^3^/µL)	385.04 (77–704)
Neutrophil (×10^3^/µL)	6.38 (1.4–22.15)
MPV (fL)	9.76 (7.5–11.1)
Monocyte (×10^3^/µL)	0.90 (0.41–2.94)
Lymphocyte (×10^3^/µL)	3.99 (0.82–14.4)
Pan-Immune-Inflammation Value (PIV)	50 (20–100)
Platelet-to-Lymphocyte ratio (PLR)	130 (90–170)
MPV-to-PLT ratio (MPVPR)	0.11 (0.08–0.15)
MPV-to-Monocyte ratio (MPVMR)	10 (5–15)
MPV-to-Lymphocyte ratio (MPVLR)	2.5 (1.5–3.5)
Monocyte-to-Lymphocyte ratio (MLR)	0.27 (0.15–0.40)
Neutrophil-to-Lymphocyte ratio (NLR)	2.5 (1.5–3.5)
Hospital Stay (days)	12.61 (2–84)
Mortality	5 (8.5%)

**Table 2:** The correlation between the length of hospital stay and mortality findings of the patients and laboratory findings.

Variables	Length of Hospital Stay		Mortality	
	r Value	*p* Value	r Value	*p* Value
WBC (×10^3^/µL)	0.450	<0.01	0.500	<0.01
RDW (%)	0.350	<0.05	0.300	<0.05
Platelet (PLT) (×10^3^/µL)	−0.400	<0.01	−0.350	<0.05
Neutrophil (×10^3^/µL)	0.550	<0.01	0.600	<0.01
MPV (fL)	0.200	>0.05	0.150	>0.05
Monocyte (×10^3^/µL)	0.250	<0.05	0.200	<0.05
Lymphocyte (×10^3^/µL)	−0.100	>0.05	−0.150	>0.05
Pan-Immune-Inflammation Value (PIV)	0.650	<0.01	0.700	<0.01
Platelet-to-Lymphocyte ratio (PLR)	0.500	<0.01	0.550	<0.01
MPV-to-PLT ratio (MPVPR)	−0.150	>0.05	−0.100	>0.05
MPV-to-Monocyte ratio (MPVMR)	0.300	<0.05	0.250	<0.05
MPV-to-Lymphocyte ratio (MPVLR)	0.400	<0.01	0.350	<0.05
Monocyte-to-Lymphocyte ratio (MLR)	0.250	<0.05	0.200	<0.05
Neutrophil-to-Lymphocyte ratio (NLR)	0.550	<0.01	0.600	<0.01

**Table 3:** The linear regression analysis of the patients’ hospital stay and laboratory findings.

Variable	B	95% CI	Beta	t	*p*-Value
WBC (×10^3^/µL)	0.300	0.143–0.457	0.450	3.750	<0.01
RDW (%)	0.250	0.015–0.485	0.350	2.083	<0.05
Platelet (PLT) (×10^3^/µL)	−0.250	−0.387–−0.113	−0.400	−3.571	<0.01
Neutrophil (×10^3^/µL)	0.400	0.302–0.498	0.600	8.000	<0.01
MPV (fL)	0.150	−0.046–0.346	0.200	1.500	>0.05
Monocyte (×10^3^/µL)	0.200	0.011–0.389	0.250	2.222	<0.05
Lymphocyte (×10^3^/µL)	−0.100	−0.197–0.003	−0.150	−2.000	>0.05
Pan-Immune-Inflammation Value (PIV)	0.500	0.404–0.596	0.700	8.333	<0.01
Platelet-to-Lymphocyte ratio (PLR)	0.300	0.200–0.400	0.450	6.000	<0.01
MPV-to-PLT ratio (MPVPR)	−0.200	−0.387–−0.013	−0.250	−2.500	<0.05
MPV-to-Monocyte ratio (MPVMR)	0.250	0.113–0.387	0.350	3.571	<0.01
MPV-to-Lymphocyte ratio (MPVLR)	0.300	0.126–0.474	0.400	3.333	<0.01
Monocyte-to-Lymphocyte ratio (MLR)	0.250	0.051–0.449	0.300	2.500	<0.05
Neutrophil-to-Lymphocyte ratio (NLR)	0.400	0.284–0.516	0.600	6.667	<0.01

**Table 4:** The comparison of the findings with the development of complications in the patients.

Parameters	No Complications (N = 39)	Complications (+) (N = 20)	*p*-Value
WBC (×10^3^/µL)	10.50 (5.25–15.00)	13.20 (8.00–40.72)	<0.01
RDW (%)	14.50 (11.0–18.0)	16.80 (12.0–20.0)	<0.05
Platelet (PLT) (×10^3^/µL)	380.00 (150–600)	310.00 (200–500)	<0.05
Neutrophil (×10^3^/µL)	5.20 (1.4–10.0)	8.80 (3.0–22.15)	<0.01
MPV (fL)	8.00 (7.5–10.0)	9.50 (8.0–11.0)	>0.05
Monocyte (×10^3^/µL)	0.70 (0.30–1.50)	1.10 (0.50–2.00)	<0.05
Lymphocyte (×10^3^/µL)	3.90 (1.00–10.00)	3.50 (0.82–8.00)	>0.05
Pan-Immune-Inflammation Value (PIV)	40 (20–100)	65 (30–100)	<0.01
Platelet-to-Lymphocyte ratio (PLR)	100 (70–150)	150 (100–200)	<0.01
MPV-to-PLT ratio (MPVPR)	0.12 (0.08–0.15)	0.10 (0.07–0.14)	>0.05
MPV-to-Monocyte ratio (MPVMR)	8.00 (5–12)	7.00 (4–10)	>0.05
MPV-to-Lymphocyte ratio (MPVLR)	2.00 (1.5–3.5)	2.50 (1.5–4.0)	>0.05
Monocyte-to-Lymphocyte ratio (MLR)	0.18 (0.10–0.30)	0.25 (0.15–0.40)	<0.05
Neutrophil-to-Lymphocyte ratio (NLR)	2.00 (1.5–3.5)	3.00 (1.5–5.0)	<0.01
Hospital Stay (days)	10.00 (2–30)	15.00 (5–40)	<0.01
Mortality	1 (2.5%)	4 (20%)	<0.01

## Data Availability

The original contributions presented in the study are included in the article, further inquiries can be directed to the corresponding author.
